# A Flare of Ulcerative Colitis Accompanied With Cerebral Sinus Venous Thrombosis And Bilateral Thalamic Infarctus: A Case Report

**DOI:** 10.4021/gr403w

**Published:** 2012-03-20

**Authors:** Ahmet Cumhur Dulger, Huseyin Begenik, Levent Demirtas, Ramazan Esen, Habib Emre

**Affiliations:** a Yuzuncu Yil University, Department of Gastroenterology, Van, Turkey; b Yuzuncu Yil University, Department of Internal Medicine, Van, Turkey; c Ipekyolu Public Hospital, Department of Internal Medicine, Van, Turkey

**Keywords:** Ulcerative colitis, Sinus vein thrombosis, Thalamic infarcts

## Abstract

Ulcerative colitis (UC) is a chronic inflammatory and recurrent disorder that is characterized by bowel inflammation. Some patients with Inflammatory Bowel Disease (IBD) have acute, severe, and sometimes devastating intracranial complications that require immediate medical intervention. Cerebral sinus vein thrombosis is a rare but serious extraintestinal complication associated with ulcerative colitis. Herein we report a 30-year-old man with UC who presented with a flare of gastrointestinal symptoms with mental obtundation and apathy. Total colonoscopy revealed active colitis and cranial MRI showed extensive cerebral sinus venous thrombosis with thalamic infarcts. Because the patient was clinically unstable metilprednisolon with low molecular weight heparin were administered. Two days after treatment the patient was died despite all medical efforts.

## Introductıon

Ulcerative colitis (UC) is a chronic inflammatory disease of the large bowel and is characterized by rectal bleeding and diarrhea [1]. UC is a result of inappropriate and ongoing activation of the mucosal immune system of large bowel. This anormal activaton is most likely due to defects in both the barrier function of the intestinal epithelium and the mucosal immune system [2].

The diagnosis of UC is generally established by endoscopic, histologic and radiologic examinations as well as laboratory tests. The medical treatment of ulcerative colitis has depend mainly on 5-aminosalicylates, corticosteroids, immunosuppressant, including thiopurine antimetabolites, and cyclosporine. These therapies are based on extent and severity of the disease [3-5].

Patients with UC commonly present with a wide range extraintestinal manifestations. Hypercoagulability-related disorders are well-recognized complications of UC. Hypercoagulability may manifest as thromboembolic events in many organ systems as well as intracranial vascular system [6].

Aseptic cerebral venous and sinus thrombosis (CVST) is characterised by headache, seizures, focal neurological deficits, altered consciousness, and papilledema [7].

## Case Report

A 30-year-old man presented to our hospital with a 2-year history of UC. He also had fever, abdominal pain with increasingly frequent bloody diarrhea, and a 2-week history of cefalgia and drowsiness that was relieved when he was taken NSAI drugs.

There was no family history of inflammatory bowel diseases. The patient’s medications included meselamine, at a dose of 1000 mg thrice daily and pantoprazol 40 mg daily. He took a nonsteroidal anti-inflammatory drug on an as-needed basis.

On physical examination, his blood pressure was 110/75 mmHg and heart rate was 90 beats per minute and regular. His temperature was 39.0 °C. There was diffuse nonspesific tenderness to palpation of abdomen. His neurologic examination revealed stupor, papilledema and right hemiplegia. Glasgow coma score was calculated as 5 point.

The hemoglobin level was 9.3 g per deciliter, with a mean corpuscular volume of 76 µm^3^. The white-cell count was 10400 per cubic millimeter, and the platelet count was 391,000 per cubic millimeter. The erythrocyte sedimentation rate was 50 mm per hour, and the C-reactive protein level was 132 mg per deciliter.

Other laboratory values were as follows: sodium, 135 mmol per liter; potassium, 3.8 mmol per liter; blood urea nitrogen, 14 mg per deciliter; creatinine, 0.7 mg per deciliter; lipase, 20 U per liter. The serum albumin level was 3.0 g per deciliter. The liver-related transaminases and cholestatic enzymes, the anticardiolipin immunoglobulin levels were also in normal ranges. The patient had a negative lupus anticoagulant and had also normal homocystein levels.

Protein S activity was 35% (normal: 60 - 150) and the antithrombin level was 45 % (normal, 85 - 125%). Stool cultures and examination of stool samples for signs of ova and parasites were also negative. A serologic test to rule out Entomoeba hystolitica was negative.

**Figure 1:**
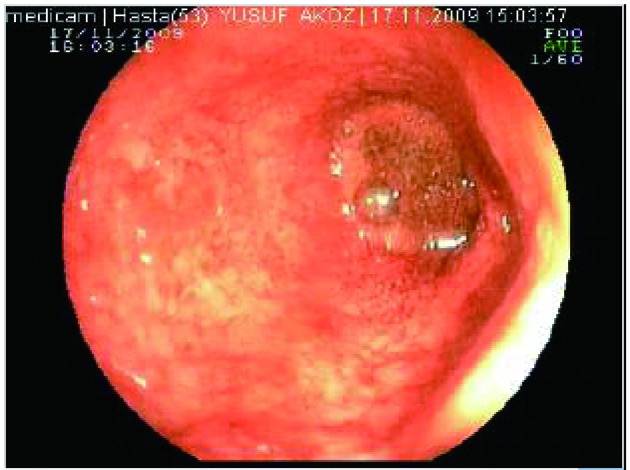
Distal colonoscopy of the patient showed mucosal hyperemia with multiple ulcers.

During the admittion, the patient underwent a limited colonoscopic examination as far as the splenic flexure and linear ulcerations were detected. The mucosal appearance was given a score of 3 which was consistent with a heavy flare of UC and five mucosal-biopsy specimens were obtained (Fig. 1). Pathologic examination of these samples was revealed UC. Chest and abdomen radiography revealed no abnormalities. Ultrasonography of the abdomen showed a thickened colonic segment, without free air.

**Figure 2:**
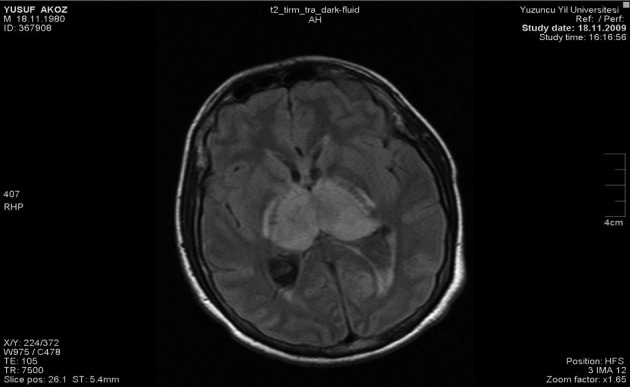
MRI of the cranium revealed infarct areas of posterior limb of capsula interna, right globus pallidus, left occipital lobe and bilateral thalamus.

**Figure 3:**
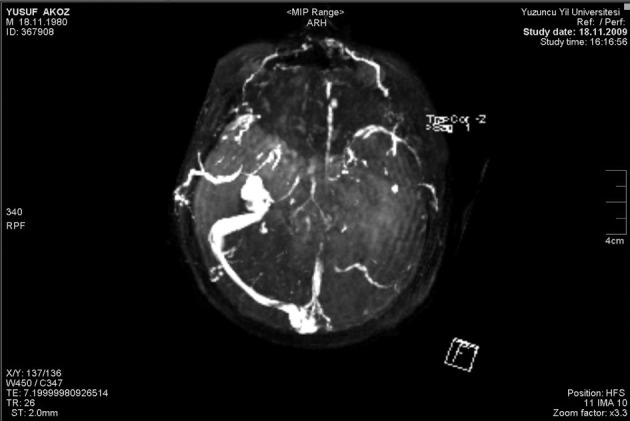
Cerebral venous MR angio showed massive thrombosis of sinus rectus, left transvers and sigmoid sinuses.

Cranial MRI showed infarct areas of posterior limb of capsula interna, right globus pallidus, left occipital lobe and bilateral thalamus (Fig. 2). Cerebral venous MR angio was consistent with thrombosis of sinus rectus, left transvers and sigmoid sinuses (Fig. 3). The patient’s ulcerative colitis and confusion and the presence of MRI findings were consistent with UC associated sinus vein thrombosis.

The patient received intravenous fluids. His treatment with mesalamine was continued, and intravenous methylprednisolon (60 mg/day) and low molecular weight heparin (LMWH) were also started. Two days after the treatment was introduced, hypotension and fixed pupils were developed and he was died in intensive care unit.

## Discussion

A patient presented in this case report manifested clinical and radiologic evidence of an acute sinus vein thrombosis in association with an acute flare of ulcerative colitis. Although UC is an intestinal disease, the outcome may depend upon a variety of extraintestinal complications, which develops overtime or suddenly [2]. As an extraintestinal complication, sinus vein thrombosis is very rare and may be fatal. The complex interactions between thrombosis and UC have not been well- characterised. But, accumulating evidence suggests that UC is a key factor in the development of thrombotic complications [8, 9].

Thrombocytosis, hyperfibrinogenemia, elevated levels of factor V, and factor VIII; antithrombin III deficiency; protein S deficiency are related to active bowel inflammation and may play a major role for thrombosis [10-14]. Recent studies showed that patients with ulcerative colitis have more frequently lower circulating protein S levels than normal controls [15, 16]. Additionaly, a reduced level of antithrombin III leading to an increased risk for thrombosis during the course of ulcerative colitis [17, 18].

However, the mechanism involved in the appearance of these coagulation abnormalities in patients with ulcerative colitis remains to be clarified [19]. Furthermore, no predisposing factor can be found in more than 50% patients with UC [9]. In our patient, the protein S activity and the level of anti-thrombin III were low and those detected abnormalities may contribute to development of sinus vein thrombosis as well as thalamic infarcts.

Defective activity of methylenetetrahydrofolate reductase also has been reported among patients with UC. It is linked to folate and vitamin B12 deficiency and causes hyperhomocysteinemia-related thrombosis [20]. In presented case, the level of homocystein was in normal ranges.

Prevalence of sinus vein thrombosis is reportedly found less than 1% among patients with inflammatory bowel disease [21, 22]. Most affected areas are reported as superior sagittal sinus and lateral sinuses [23]. However, association of thalamic infarcts with sinus vein thrombosis has rarely reported in English literature [24].

Despite the ideal treatment of ulcerative colitis- related sinus vein thrombosis is not well-established, Low Molecular Weight Heparin (LMWH) is most preferred agent to treat of this devastating complication [14] but despite all efforts, clinical endpoint of the discussed case was worst due to progressive intracranial complications.

Taken together, to determine and to prevent intracranial complications, clinicians should remain vigilant in patients with UC those have neurologic symptoms. Furthermore, neurologic symptoms should routinely be checked by clinicians during acute attacks of ulcerative colitis.
